# Glaucocalyxin A Inhibits the Malignancies of Gastric Cancer Cells by Downregulating MDM2 and RNF6 *via* MiR-3658 and the SMG1-UPF mRNA Decay Pathway

**DOI:** 10.3389/fonc.2022.871169

**Published:** 2022-06-22

**Authors:** Yanqi Liu, Ping Chen, Daqing Qi, Linhui Chen

**Affiliations:** ^1^ Department of Gastroenterology, The Affiliated Hospital of Inner Mongolia Medical University, Hohhot, China; ^2^ Department of Medical Affairs, Hangzhou Huqingyu Hall Pharmaceutical Co., Ltd., Hangzhou, China

**Keywords:** gastric cancer, Glaucocalyxin A, malignancy, MDM2, RNF6, miR-3658, mRNA decay

## Abstract

Gastric cancer (GC) ranks as the most common gastrointestinal cancer and is among the leading causes of cancer death worldwide. Glaucocalyxin A (GLA), an entkauranoid diterpene isolated from Rab-dosia japonica var., possesses various bioactivities. To date, the data on the effect of GLA on GC are still minimal, and the molecular mechanisms remain largely unknown. Herein, we found that GLA could significantly inhibit the proliferation, cell adhesion, and invasion of HGT-1, SNU-1, SNU-6, and NCI-N87 GC cells in a dose-dependent manner. GLA enhanced the apoptosis of the GC cells as evidenced by the increased caspase-3 activity and the elevated levels of cleaved caspase-3 and cleaved PARP in GC cells in the presence of GLA. We then showed that the downregulation of Murine Double Minute Clone 2 (MDM2) and Ring Finger Protein 6 (RNF6) by GLA was implicated in the GLA-induced inhibition of the GC cells. Furthermore, MDM2 and RNF6 were identified as the targets of miR-3658 that was downregulated in the GC cells and upregulated by GLA. Moreover, it was shown that miR-3658 was hypermethylated in the GC cells, and GLA could rescue the expression of miR-3658 *via* demethylation by abrogating EZH2-mediated epigenetic silencing. In addition to the miR-3658-MDM2/RNF6 regulatory axis, activation of the SMG1-UPF mRNA decay pathway contributed to the downregulation of MDM2 and RNF6 by GLA in the GC cells. The inhibitory effect of GLA on gastric cancer and the expression of MDM2 and RNF6 was also validated in *in vivo* study. Our findings suggest that has the therapeutic potential for GC by downregulating oncogenes *via* posttranscriptional regulation.

## Introduction

GC is the most common gastrointestinal cancer and a leading cause of cancer death worldwide (1). Despite the tremendous efforts and progress made in recent decades in treating GC, the overall survival rate for patients remains poor. Therefore, it is urgent to discover and develop new therapeutic approaches or agents for GC. Glaucocalyxin A (GLA), an entkauranoid diterpene isolated from Rab- dosia japonica var., exhibits multiple pharmacological effects such as anticancer effect, induction of osteogenesis, suppression of inflammation, and prevention of myocardial infarction (2–9). MDM2 and RNF6 are extensively-studied oncoproteins in various malignancies, including GC (10–13). *Mdm2* is an oncogene mapped to chromosome 12q13-14, and the most critical function of MDM2 in cancers is to target the tumor suppressor P53 protein *via* proteasomal degradation or blockade of activity (10). MDM2 protein is so far the best negative modulator of P53. MDM2 can enhance tumor onset and progression by catalyzing P53 ubiquitination that induces degradation and deactivating the transactivation domain of P53 (10). *Rnf6* is located on chromosome 13q12.13, and as a tumor promoter, RNF6 belongs to the E3 ligase family and is implicated in the growth, proliferation, adhesion, and invasion of cancer cells *via* the ubiquitination process. RNF6 can regulate the activation of the Wnt/β-catenin pathway and JAK/STAT3 pathways in cancer (14, 15).

MicroRNAs (miRNAs) are small single-stranded non-coding RNA molecules containing 19-23 nucleotides that have emerged as posttranscriptional regulators of the target genes (16, 17). MiRNAs target downstream genes by interacting with the specific binding site in the three prime untranslated regions (3’-UTR) in mRNA. MiRNAs have been documented to be involved in GC initiation and progression, and the pattern of deregulated miRNAs represents the features related to diagnosis, prognosis, and therapeutic resistance in GC (16–19). Recent data have revealed that miR-3658 acts as tumor suppressor miRNA in colorectal cancer by targeting octamer-binding transcription factor 4 (Oct4) (17). In addition to deregulated RNA silencing by miRNAs that occurred at the posttranscriptional level in cancer cells, the impairment of the nonsense-mediated mRNA decay (NMD) mechanism plays a critical role in driving the onset and progression of cancer *via* posttranscriptional regulation (20).

To date, the data on the effect of GLA on GC are still minimal, with molecular mechanisms yet to be extensively revealed. A recent study conducted by Zhou et al. showed that GLA could prevent hypoxia-induced epithelial-mesenchymal transition in MGC-803 GC cells *via* the PI3K/Akt signaling pathway (21). Herein, we found that GLA can inhibit the malignant behaviors of GC cells by downregulating the expression of MDM2 and RNF6 *via* epigenetic abrogation of EZH2-mediated miR-3658 silencing. Moreover, GLA can also suppress the expression of MDM2 and RNF6 by enhancing mRNA decay through activation of the SMG1-UPF mechanism.

## Materials and Methods

### Materials

**Figure 1:**
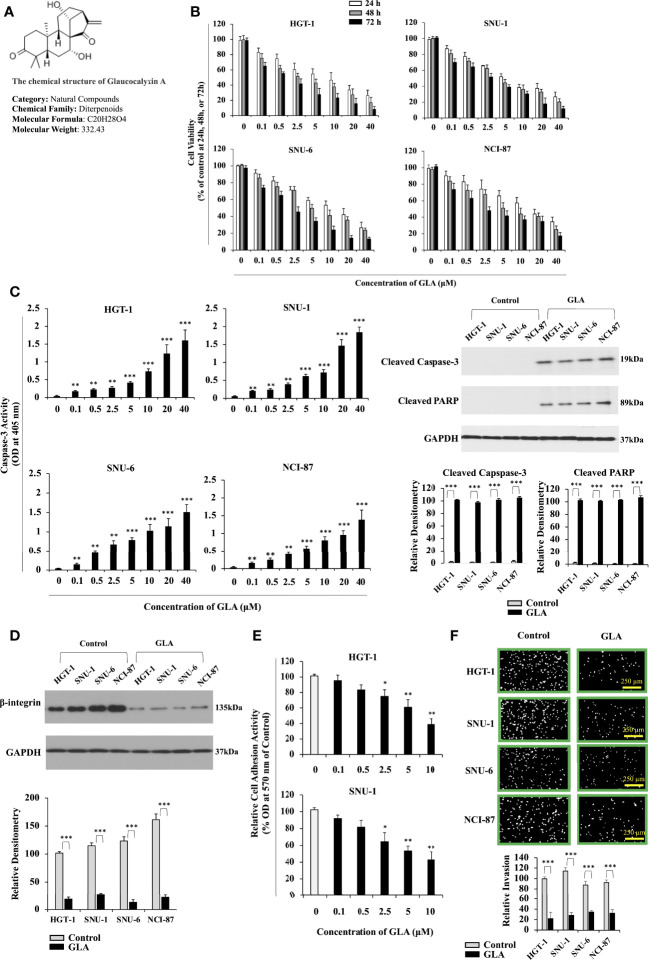
Inhibition of the malignant behavior of GC cells by GLA. **(A)** Chemical structure of GLA. **(B)** Changes in cell viability of HGT-1, SNU-1, SNU-6, and NCI-N87 GC cell lines treated with 0, 0.1, 0.5, 2.5, 5, 10, 20, and 40 µM of GLA at 24 h, 48 h, or 72h (% of control). **(C)** The caspase-3 activity was shown as OD values at 405 nm in HGT-1, SNU-1, SNU-6, and NCI-N87 treated with the indicated concentrations of GLA (left panel). Western blot analysis of cleaved caspase-3 and cleaved PARP in HGT-1, SNU-1, SNU-6, and NCI-N87 cells in the presence of 10 µM of GLA (right panel). GAPDH served as an internal control. **(D)** Western blot analysis of β-integrin in HGT-1, SNU-1, SNU-6, and NCI-N87 cells in the presence of 10 µM of GLA. **(E)** Cell adhesion activity shown as OD values at 570 nm in HGT-1 and SNU-1 cells in the presence of 0, 0.1, 0.5, 2.5, 5, or 10 µM of GLA for 90 min. **(F)** Invasion of HGT-1, SNU-1, SNU-6, and NCI-N87 cells treated with 10 µM of GLA of for 12 h (100 × magnification; scale bar-250 µm). The Western blot analysis was repeated three times. The data represented the mean ± SD (n=3). *p < 0.05, **p < 0.01, ***p<0.001.

GLA (**Figure 1A**) is a natural compound obtained from BOC Sciences, New York, USA, with white powder appearance and >98% purity. The molecular formula of GLA is C20H28O4, and the molecular weight of 332.43 Da. GLA was dissolved in dimethyl sulfoxide (DMSO). Human SNU-1, SNU-16, NCI-N87, normal gastric epithelial GES-1 cells, and Hs 738.St/Int cells were from ATCC, Virginia, USA. RPMI-1640 medium, Dulbecco’s Modified Eagle’s Medium (DMEM), fetal bovine serum (FBS), 100 U/ml penicillin and streptomycin, miR-3658 inhibitor, Lipofectamine LTX transfection reagent, 4% paraformaldehyde, SMG1 siRNA and control, Pierce BCA protein assay kit, and IL-1β and TNF-α ELISA kits were obtained from Thermo Fisher Scientific, Massachusetts, USA. Human HGT-1 cells, Matrigel, fibronectin, 5-Aza-CdR, miR-3658 mimic, lysis buffer, PVDF membrane, ECL detection reagent, EZ-Magna CHIP kit, actinomycin D, and N-Methyl-N-nitrosourea (MNU) were obtained from MilliporeSigma, Massachusetts, USA. Matrigel-coated Transwell inserts (8 μm: pore size) and cell culture plates were obtained from Corning, New York, USA. Primary antibodies used in the present study include rabbit anti-cleaved caspase-3 antibody, rabbit anti-cleaved PARP antibody, Rabbit anti-β-integrin antibody (Cell Signaling Technology, Massachusetts, USA), rabbit anti-MDM2 antibody, rabbit anti-RNF6 antibody, rabbit anti-GAPDH antibody, rabbit anti-EZH2 antibody, and rabbit anti- H3K27me3 antibody (Abcam, Massachusetts, USA). Goat-anti rabbit IgG, rabbit normal IgG, and WST-8/CCK8 kit were obtained from Abcam. Caspase-3 colorimetric assay kit was obtained from BioVision (California, USA). Human MDM2 and RNF6 lentivirus particles were from Creative Biogene, New York, USA. Trizol and SuperScript IV first-stand Synthesis System were from Invitrogen, Massachusetts, USA. TaqMan MicroRNA Reverse Transcription Kit, U6 snRNA, and PowerUp SYBR Green Master Mix PCR kit were from Applied Biosystems, Massachusetts, USA. Myeloperoxidase (MPO) assay kit was the product from HyCult Biotechnology, Uden, The Netherlands.

### Cell Culture

Human GC cell lines HGT-1, SNU-1, SNU-16, and NCI-N87 were maintained in RPMI-1640 medium supplemented with 10% FBS and 100 U/ml penicillin and streptomycin at 37°C in a humidified incubator with 5% CO_2_. Normal human gastric epithelial GES-1 cells and Human Hs 738.St/Int cells were cultured in DMEM supplemented with 10% and FBS and 100 U/ml antibiotics.

### Cell Counting Kit 8 (WST-8/CCK8) Assay

Cell viability was examined using the WST-8/CCK8 kit according to the manufacturer’s instruction. Briefly, the GC cells were seeded at 5×10^3^ cells/well in 96-well culture plates. After 3 hours of recovery, the cells were treated with serial concentrations of GLA for 24 h, 48 h, or 72 h. Subsequently, WST-8/CCK8 solution was added to each well and incubated for 4 hours at 37°C. WST-8/CCK8 tetrazolium salt was reduced by cellular dehydrogenases to a formazan product soluble in culture medium. The amount of formazan produced was proportional to the number of living cells and was measured by absorbance at 460 nm using a BioTek microplate reader.

### Western Blot Analysis

GC cells with different treatment were lysed in lysis Buffer. Gastric tumor tissues from mice were lysed with lysis buffer using a Miltenyi gentleMACS Dissociator. Protein concentrations were determined using BCA protein assay kit. 30-50 μg of total protein was subjected to 4-10% SDS-PAGE gels, transferred onto PVDF membranes, and blocked in 5% non-fat milk for 1 h at room temperature. The membranes were then incubated with specific primary antibodies overnight at 4°C. The membranes were subsequently incubated with secondary antibodies conjugated with horseradish peroxidase for 1 h at room temperature. The immunoblots were subsequently visualized using ECL detection reagents. The densitometry of the bands was analyzed using Image J.

### Cell Adhesion Assay

For the cell adhesion assay, a 96-well plate was pre-coated with 300 µg/mL of Matrigel and 10 μg/mL fibronectin at 4°C overnight and blocked with 1% (w/v) albumin. GC cells were centrifuged, resuspended in complete medium, and seeded on the 96- well plate at a density of 1 × 104 per well. After adhering for 90 min, the cells in the wells were washed with phosphate-buffered saline (PBS), fixed with 4% paraformaldehyde for 10 min, and stained with 0.5% crystal violet for 10 min. The stained attached cells were lysed using 30% glacial acetic acid for 10 min. OD values at 570 nm were then measured.

### Cell Invasion Assay

For the cell invasion assay, 2 × 10^4^ GC cells (100 μl per well) were seeded in a Matrigel-coated Transwell insert (8 μm: pore size) in a 24-well culture plate and maintained in serum-free medium. The medium with different concentrations of GLA was added to the lower chamber. After incubation for 12h at 37°C, cells on the top surface of the insert were removed with a cotton swab, and cells that invaded to the bottom surface of the insert were fixed with 4% paraformaldehyde for 30 minutes, stain Transwell inserts in DAPI for 10 minutes then subjected to microscopic inspection.

### Transfection

HGT and SNU-1 cells reaching 70% confluence in a six-well culture were transfected with 10 pM of miR-3658 inhibitor, mimic, or the negative controls, and 2 µg of PGL3 luciferase reporter vectors containing either wild-type 3’ UTR or muted 3’UTR of MDM2 or RNF6 using Lipofectamine LTX reagent according to the manufacturers’ instructions. The culture medium was replaced with complete medium at 6 h after transfection. The cells were harvested for subsequent assays after 48 h transfection.

### RNA Extraction and Reverse Transcription Quantity (RT- PCR)

Total RNA was extracted from the GC cells and gastric tumor tissues from mice using Trizol. 5 ng of purified RNA were retro-transcribed using TaqMan MicroRNA Reverse Transcription Kit. The levels of miR-3658 were measured using Real-Time quantitative PCR according to the TaqMan MicroRNA Assay protocol, and U6 snRNA served as an internal control. The PCR reaction was carried out in a 96-well PCR microplate at 95°C for 10 min followed by 40 cycles of 95°C for 15 s and 60°C for 1 min. The primers for miR-3658 are forward 5’-GTGGGGGTTTAAGAAAACACCAT-3’ and reverse 5’- GTGCAGGGTCCGAGGT-3’. To analyze the mRNA levels of MDM2 and RNF6, 1 μg/ml of total RNA was reversely transcribed into cDNA using a SuperScript IV first-stand Synthesis System. Real-Time PCR was then performed using a PowerUp SYBR Green Master Mix PCR kit. The PCR reactions were performed at 95°C for 15 min, followed by 40 cycles of 95°C for 30 s and 58°C for 30 s. The primers for human MDM2, RNF6, and the internal control 18S were as follows: MDM2 (human) forward 5’-CACCTCACAGATTCCAGCTT-3′ and reverse 5′- CGCCAAACAAAT CTCCTAGA-3′; MDM2 (mouse) forward 5’- ATGAGGTCTATCGGGTCACAGT-3′ and reverse primer 5′-CACATCCAAG CCTTCT TCTGC-3′; RNF6 (human) forward 5’- AGAAGATGGCAGCAAGAGCG -3’ and reverse 5’- TCAAGTCAGGCTGAGATGCTAGT-3’;18S (human) forward 5’- AGCAGCCGCGGTAAT TCCAGCT -3’and reverse 5’- CGGGACACTCAGCTAAGAGCATC-3’; 18S (mouse) forward 5’- AGTCCCTGC CCTTTGTACACA -3’ and reverse 5’- CGATCCGAGGGCCTCACTA-3’. Primers for mouse RNF6 were the products of Amsbio, Cambridge, USA. The PCR results were calculated using the 2-ΔΔCt method.

### Chromatin Immunoprecipitation (CHIP) Assay

CHIP assays were performed using an EZ-Magna CHIP kit according to the manufacturer’s instructions. After being fixed with 1% formaldehyde, nucleoprotein complexes were sheared to 200–500 bp with sonication and then immunoprecipitated with anti-EZH2 or anti-H3K27me3 antibodies overnight at 4°C. The enrichment of the DNA fragment of miR-3658 upstream coding region in the immunoprecipitated complexes was then detected using PCR.

### Statistical Analysis

All the data are shown as mean ± standard deviation (S.D). Statistical significance was determined by unpaired Student’s two-tailed *t*-test or one-way ANOVA analysis, with *p* < 0.05 was considered to be significant (*p < 0.05, **p < 0.01, ***p < 0.001). Incidences in the *in vivo* study were assessed by Chi-square test, followed by pairwise analysis. Each experiment was performed a minimum of three times.

### Animal Experiments

Animal experiments were performed according to the guidelines provided by the experimental animal center of Hangzhou Huqingyu Hall Pharmaceutical Co., Ltd, Hangzhou, China, and approved by the Animal Care and Use Committee of Hangzhou Huqingyu Hall Pharmaceutical Co., Ltd. 24 six-week-old C57BL/6 mice were divided into 4 groups with 6 mice in each group: vehicle control, *H. pylori* + MNU, GLA only*, H. pylori* + MNU+ GLA*. H. pylori* and MNU were used to induce gastric cancer in the mice. The mice were inoculated with a total dose of 1 × 108 colony-forming units *of H. pylori (*Sydney strain 1) per mouse in 200 μl of trypticase broth by oral gavage 6 times for 2 weeks before MNU administration. The mice were given MNU freshly dissolved in distilled drinking water at the concentration of 240 ppm in a light-shielded bottle on alternate weeks for 10 weeks. The mice were then kept being housed with free access to food and water, waiting for the development of gastric tumors for 18 weeks. 10 mg/kg GLA were administrated by oral gavage twice a week for 12 weeks after 6 weeks post MNU treatment. The mice were sacrificed by CO_2_ asphyxiation and stomach tissue was taken for the subsequent process at 18 weeks after completion of MNU induction. To examine the pro-inflammatory status in the gastric tissues by measuring the levels of mucosal myeloperoxidase (MPO), a marker of inflammation, and pro-inflammatory cytokines, 20 mg of scraped gastric mucosa from mice was homogenized in 500 μL of lysis buffer on ice and centrifuged at 13,000 rpm for 15 minutes. The supernatant was collected for MPO assay and the ELISA for IL-1β and TNF-α according to the manufacturers’ instructions.

## Results

### GLA Inhibits the Malignant Behavior of GC Cells

We sought to investigate the effect of GLA on the malignant behaviors of GC cells. HGT-1, SNU-1, SNU-6, and NCI-N87 GC cell lines were treated with 0, 0.1, 0.5, 2.5, 5, 10, 20, and 40 µM of GLA for 24 h, 48 h, or 72h. We found that GLA exerted an antiproliferative effect on all four GC cell lines in a dose-dependent fashion (**Figure 1B**) but didn’t significantly affect the cell viability of normal human gastric epithelial GES-1 cells and human Hs 738.St/Int cells at the concentrations from 0.1 µM to 10 µM (**Supplementary Figure 1**). Thus, 10 µM was set as the highest concentration of GLA in our mechanistic assays. We examined the effect of GLA on the apoptotic status of the GC cells. The caspase-3 activity and the expression of cleaved caspase-3 and cleaved PARP were significantly increased by GLA treatment (**Figure 1C**). Collectively, the GC cells exhibited a greatly reduced ability to proliferate, accompanied by dramatically improved apoptosis in the presence of GLA. Cell adhesion is among the essential steps for cancer cells to invade. We further examined the impact of GLA on the cell adhesion process in the GC cells. The expression of β1-integrin, a critical protein associated with cell adhesion, was downregulated in the presence of GLA (**Figure 1D**). As shown in **Figure 1E**, GLA significantly blocked the adhesion of GC cells to fibronectin and Matrigel in a dose-dependent manner. Consistently, the invasive capability of the GC cells was also hampered by GLA (**Figure 1F**).

### The Inhibitory Effect of GLA on GC Cells Is Mediated by Downregulating MDM2 and RNF6

**Figure 2:**
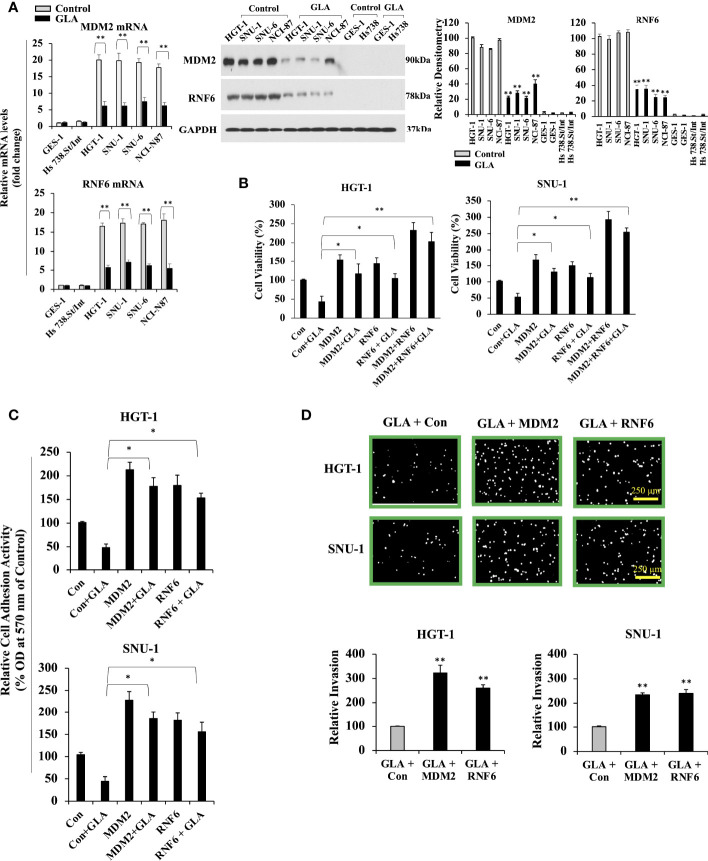
Inhibition of MDM2 and RNF6 in GC cells by GLA. **(A)** Fold change in mRNA levels of MDM2 and RNF6 examined by real-time RT-PCR in GES-1, Hs 738. St/Int, HGT-1, SNU-1, SNU-6, and NCI-N87 cells with the treatment of 10 µM of GLA (left panel). Western blot analysis of MDM2 and RNF6 in the indicated GC cells treated with GLA (right panel). **(B)** Changes in cell viability of HGT-1 and SNU-1 cells with restored overexpression of MDM2 or RNF6 in the presence of 10 µM of GLA for 48 h. **(C)** Cell adhesion activity and invasion (100 × magnification; scale bar-250 µm) **(D)** of HGT-1 and SNU-1 cells with restored overexpression of MDM2 or RNF6 in the presence of 10 µM of GLA for 12 h. The Western blot analysis was repeated three times. The data represented the mean ± SD (n=3). *p < 0.05, **p < 0.01 vs control.

*Mdm2* and *RNF6* are classified as oncogenes in various types of cancer, including gastric cancer. Our previous screening of the genes that are regulated by GLA in gastric cancer cells revealed that *Mdm2* and *RNF6* were the most downregulated genes, among others, in response to GLA treatment in gastric cancer cells. Thus, we selected *Mdm2* and *RNF6* in the present study to explore the molecular mechanism underlying the inhibitory effect of GLA in gastric cancer cells. We explored the role played by GLA-MDM2/RNF6 in controlling the malignant behavior of GC cells. As shown in **Figure 2A**, the expression of MDM2 and RNF6 were dramatically upregulated in all the four GC cells at mRNA and protein levels, and GLA significantly inhibited the expression of MDM2 and RNF6. We then explored the relationship between MDM2 and RNF6 expression and GLA effect. Given the consistent results in the four gastric cell lines in the presence of GLA, we then chose and focused on two cell lines (HGT-1and SNU-1) among them for further mechanistic studies. We found that restoration of MDM2 and RNF6 by ectopic overexpression could significantly abrogate the inhibitory effect of GLA on proliferation (**Figure 2B**), adhesion (**Figure 2C**), and invasion (**Figure 2D**) of the HGT-1 and SNU-1 cells.

### MDM2 and RNF6 Are the Targets of MiR-3658 That Is Downregulated in GC Cells and Upregulated by GLA Treatment

**Figure 3:**
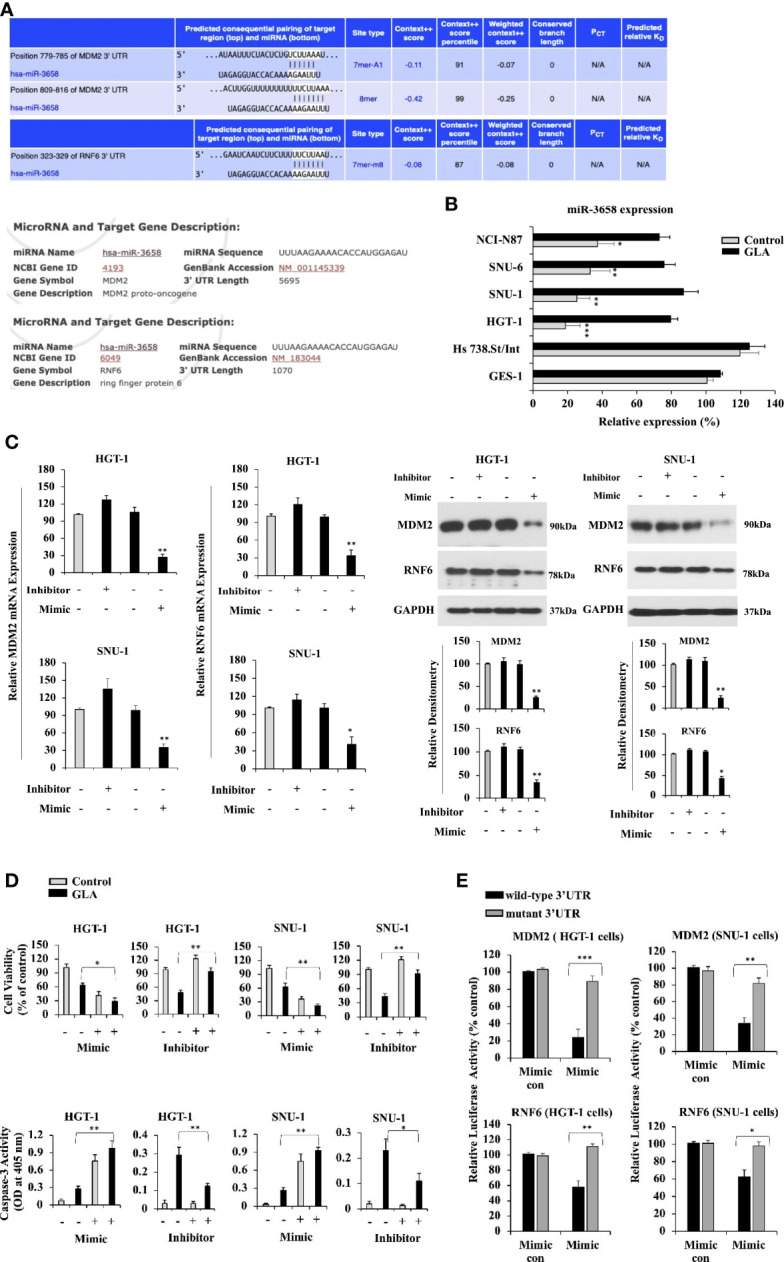
MDM2 and RNF6 are the targets of miR-3658 that is upregulated by GLA in GC cells. **(A)** MDM2 and RNF6 were predicted as the targets of miR-3658 using prediction algorithms, TargetScan and miRDB. **(B)** miR-3658 expression in GES-1, Hs 738.St. Int, HGT-1, SNU-1, SNU-6, and NCI-N87 cells with or without GLA treatment (10 µM). **(C)** The effects of miR-3658 inhibitor and mimic on the expression of MDM2 and RNF6 at mRNA (left panel) and protein levels (right panel) and on **(D)** proliferation (upper panel) and apoptotic status (caspase-3 activity) (lower panel) of HGT-1 and SNU-1 cells. **(E)** Changes in the luciferase activity in HGT-1 and SNU-1 co-transfected with PGL3 reporter vector containing either wild-type 3’ UTR or muted 3’UTR of MDM2 or RNF6 and miR-3658 mimic or mimic control. The Western blot analysis was repeated three times. The data represented the mean ± SD (n=3). *p < 0.05, **p < 0.01, ***p < 0.001.

It has been reported that microRNAs play important roles in regulating the expression of oncogenes. To confirm whether microRNAs are involved in inhibiting the expression of MDM2 and RNF6 by GLA, TargetScan and miRDB prediction algorithms were utilized to predict the microRNAs targeting MDM2 and RNF6. MDM2 and RNF6 were both predicted targets of miR-3658 (**Figure 3A**). Compared to the levels of miR-3658 expressed in GES-1 normal gastric epithelial cells and Hs 738.St/Int cells, miR-3658 was significantly downregulated in GC cells (**Figure 3B**). MiR-3658 was upregulated in GC cells in response to GLA treatment (**Figure 3B**). MiR-3658 mimic could dramatically downregulate the expression of MDM2 and RNF6 at mRNA and protein levels in the GC cells (**Figure 3C**). Thus, we deduced that miR-3658-MDM2/RNF6 might be implicated in the inhibition of GC cells by GLA. It was found that overexpression of miR-3658 by transfection of its mimic enhanced the effect of GLA, whereas the inhibitor of miR-3658 mitigated the effect of GLA in GC cells (**Figure 3D**). To further validate whether miR-3658 directly targets MDM2 and RNF6, a PGL3 reporter vector containing either wild-type 3’ UTR or muted 3’UTR of MDM2 or RNF6 was performed. As shown in **Figure 3E**, the fluorescent reporter intensity was decreased in the cells with transfection of wild-type 3’UTR of MDM2 or RNF6 but remained unaffected in the cells transfected with mutant 3’UTRs in the GC cells transfected with miR-3658 mimic. These findings suggest that miR-3658 silences MDM2 and RNF6 by directly targeting.

### MiR-3658 Is Hypermethylated in GC Cells, and GLA Can Restore MiR-3658 by Abrogating EZH2-Mediated Epigenetic Silencing

**Figure 4:**
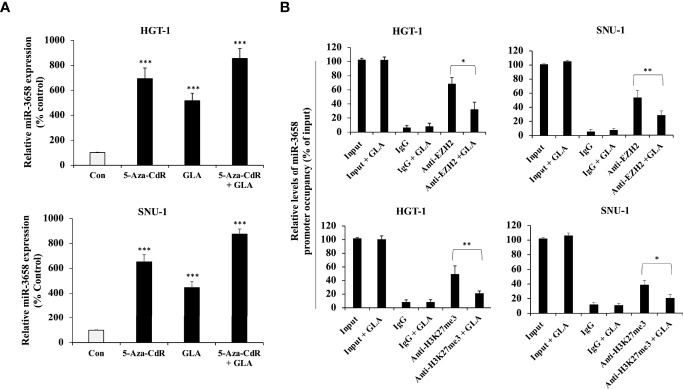
Epigenetic regulation of miR-3658 by GLA. **(A)** HGT-1 and SNU-1 cells were treated with 5 μmol/L of 5-Aza-CdR for 72 h to induce demethylation in the presence of GLA (10 µM) or in the absence of GLA. **(B)** CHIP assay for the effect of GLA on the occupancy of EZH2 and histone methylation marker H3K27me3 to the upstream region of miR-3658. The data represented the mean ± SD (n=3). *p < 0.05, **p < 0.01, ***p < 0.001.

Since miR-3658 could act as a tumor suppressor microRNA and is downregulated in gastric cancer, we sought to explore the mechanism underlying the restoration of miR-3658 by GLA in GC cells. Cancer-related aberrant methylation is commonly observed in miRNA genes, and the hypermethylation leads to the downregulation of the expression of tumor-suppressive miRNAs in cancer cells (22–25). To investigate whether DNA methylation is involved in the downregulation of miR-3658 in GC cells, we treated GC cells with 5-Aza-CdR, an S-phase-specific demethylating agent, to induce demethylation. As shown in **Figure 4A**, the levels of miR-3658 were significantly elevated in the cells treated with 5-Aza-CdR or GLA, suggesting that miR-3658 is hypermethylated in GC cells, and GLA may increase miR-3658 expression *via* demethylation. EZH2 is the enzymatic subunit of Polycomb Repressive Complex 2 (PRC2) that functions to silence the transcription of target genes by hyper-methylating histone H3 lysine 27. As shown in **Figure 4B**, EZH2 could directly bind to the upstream region of miR-3658, and the occupancy of histone methylation H3K27me3 was also detected in the CHIP precipitate. In contrast, GLA treatment blocked the association of EZH2 and H3K27me3 with the upstream region of miR-3658. These findings indicate that GLA could restore miR-3658 expression by abrogating EZH2-mediated epigenetic silencing through hypermethylation.

### GLA Activated the SMG1-UPF mRNA Decay Mechanism, Which Mediated mRNA Decay of MDM2 and RNF6

**Figure 5:**
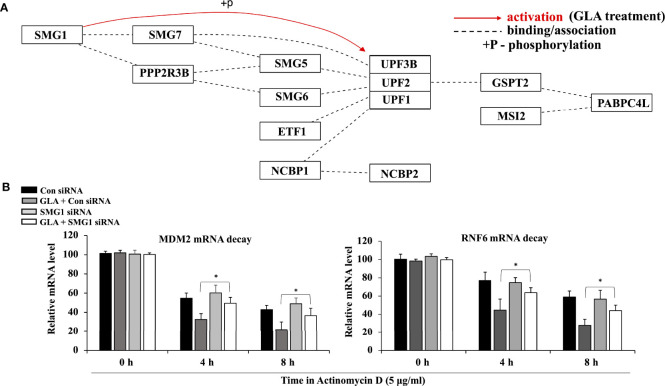
Activation of the SMG1-UPF mRNA decay mechanism by GLA contributes to the downregulation of MDM2 and RNF6 in GC cells. **(A)** Pathway analysis of SMG1-UPF in GC cells in the presence of GLA. **(B)** The stability of MDM2 and RNF6 mRNA at 0 h, 4 h, and 8 h in HGT-1 and SNU-1 cells with SMG1 siRNA transfection after treatment with 5 µg/ml of actinomycin D and 10 µM of GLA. The data represented the mean ± SD (n=3). *p < 0.05.

SMG1-UPF mechanism functions to downregulate the expression of various essential factors that facilitate tumorigenesis by inducing their mRNA decay (26, 27). Our analysis of pathway-based sequencing data integration and visualization conducted and generated using RNA from GC cells treated with GLA performed by Arraystar Inc. showed that GLA could activate the SMG1-UPF3B pathway by phosphorylation (**Figure 5A**). To investigate the effect of GLA on the mRNA stability of MDM2 and RNF6 in gastric tumor cells, we used actinomycin D, a transcription inhibitor widely used to inhibit the synthesis of new mRNA in the assessment of mRNA decay. We found that GLA significantly enhanced mRNA decay of both MDM2 and RNF6 in HGT-1 and SNU-1 cells (**Figure 5B**). Given that the generation of new mRNAs of MDM2 and RNF6 was blocked by actinomycin D, the decrease in the levels of the mRNAs in GLA treated cells reflected an accelerated mRNA degradation compared to that in control cells in the absence of GLA. Furthermore, silencing the expression of SMG1 by siRNA led to the reduction in the mRNA decay enhancement triggered by GLA treatment (**Figure 5B**). A significant difference between control siRNA and SMG1 siRNA control was not observed in our study, which is consistent with the fact that the activation or function of SMG1-UPF3B by phosphorylation is closely involved in GLA-accelerated decay of MDM2 and RNF6 mRNAs and the SMG1-UPF3B pathway is inactivated in gastric cancer cells in the absence of GLA. Thus, our results indicate that GLA could inhibit the expression of MDM2 and RNF6 in GC cells *via* not only upregulation of miR-3658 but also activation of the SMG1-UPF mRNA decay mechanism.

### GLA Alleviates Gastric Cancer and Downregulates the Expression of MDM2 and RNF6 *In Vivo*

**Figure 6:**
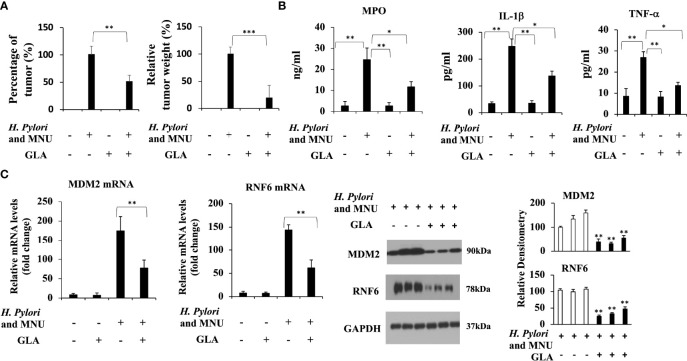
The inhibitory effect of GLA in the *in vivo* gastric cancer mouse model. **(A)** The incidence and relative tumor weight of gastric tumors at 18 weeks after MNU induction. The incidence was calculated as the percentage of tumor-bearing mice/total mice in each group. **(B)** The mucosal levels of MPO and pro-inflammatory cytokines, IL-1β and TNF-α in group, were examined at 18 weeks after MNU induction. **(C)** The expression of MDM2 and RNF6 at mRNA and protein levels in stomach tissues in each group. The Western blot analysis was repeated three times. The data represented the mean ± SD (n=3). *p < 0.05, **p < 0.01, ***p < 0.001.

We then sought to determine whether GLA could inhibit gastric cancer induced by a combination of *H. pylori* and MNU in mice. The effective number of mice and the incidences of gastric tumors are shown in **Table 1** and **Figure 6A**. The incidences and tumor weight were significantly decreased in GLA treated group. No gastric tumors were formed in the negative control group and GLA only group. The levels of mucosal MPO and the expression of pro-inflammatory cytokines, IL-1β and TNF-α in gastric mucosal were examined to evaluate the pro-inflammatory status. As shown in **Figure 6B**, the gastric mucosal MPO levels were significantly increased in *H. pylori* and MNU group compared to that of the negative control group and GLA only group, which was reduced by GLA treatment. The changes in the levels of IL-1β and TNF-α were consistent with the MPO result. Furthermore, the expression of MDM2 and RNF6 was increased in the tumor tissues in *H. pylori* and MNU group, and the effect was abrogated by GLA treatment (**Figure 6C**). There was no detectable MDM2 and RNF6 expression in the control group and GLA only group (data not shown).

**Table 1:** Incidence of gastric tumors in the *in vivo* study.

Group	Effective number	Incidence (%)Total tumor
Negative control	0	0
*H. pylori* + MNU	6	100
GLA	0	0
*H. pylori* + MNU + GLA	3	50*

Incidences were assessed by Chi-square test, followed by pairwise analysis.

^*^P<0.05 vs H. pylori + MNU group.

## Discussion

Despite the advancements in the treatment of GC, the 5-year overall survival of GC remains low. The discovery and development of new molecular compounds for treating GC and exploring the underlying molecular mechanisms are essential for developing novel therapeutic regimens that improve GC prognosis. In the present study, we revealed the GLA-MDM2/RNF6 inhibitory mechanism underlying the suppressive effect of GLA on GC. The studies on the role of GLA and the mechanism in inhibiting GC remain minimal. In the present study, we found that GLA significantly inhibited the malignant behavior of GC cells, and the inhibitory effect of GLA on GC cells was mediated by downregulating MDM2 and RNF6. The downregulation of MDM2 and RNF6 by GLA was at least by restoring miR-3658 and inducing SMG1-UPF-MDM2/RNF6 mRNA decay machinery.

MDM2 inhibits the transactivation of tumor suppressor P53. Being a P53-specific E3 ubiquitin ligase, MDM2 is a principal factor in P53 destruction (28). MDM2 has proved to play an essential role in cancer progression by inducing cell proliferation, invasion, and therapeutic resistance and inhibiting the apoptotic process, serving as a useful predictive marker for poor prognosis in various human cancers such as GC, breast cancer, hepatocellular carcinoma, and lung cancer (11, 29–31). MDM2 expression parameters are closely correlated with *H. pylori* infection and higher stages GC (29). We found that GLA treatment dramatically inhibited the expression of MDM2, suggesting that the reduction of MDM2 levels in the presence of GLA is involved in hampering the malignant behavior of GC cells. RNF6, a RING-type E3 ubiquitin ligase, has been found to play an oncogenic role in cancer (15, 32, 33). RNF6 belongs to the RNF family, which induces proteasomal degradation of its downstream proteins by ubiquitination and tagging (34–36). Similar to MDM2, RNF6 is also downregulated by GLA in GC cells. Given the E3 ubiquitin ligase property of both MDM2 and RNF6 in cancer metabolism (37), GLA might exert its antitumor effect on GC cells by blocking MDM2 and RNF6-mediated deregulated ubiquitination processes.

We then sought to investigate how GLA downregulates MDM2 and RNF6 expression in GC cells. The roles played by miRNAs, either as a tumor suppressor or as tumor enhancer, in regulating oncogene expression in cancers have been widely reported (38–40). MiR-3658 was then introduced as a bona fide upstream negative regulator for both MDM2 and RNF6 by prediction algorithms, which was validated by the inverse correlation in expression between miR-3658 and MDM2/RNF6 and the direct interaction in the *in vitro* assay. Hosseini et al. have shown that miR-3658 acts as a tumor suppressor in colorectal cancer cells. MiR-3658 was downregulated and the forced overexpression of miR-3658 in colorectal cancer cells brought about proliferation inhibition, cell cycle arrest, and retarded mobility (17). Consistent with their findings, we found that miR-3658 was downregulated in GCs and functioned to correct the malignant behaviors of GC cells. Furthermore, the levels of miR-3658 were elevated in response to GLA treatment in GC cells, suggesting that restoration of the function of miR-3658-MDM2/RNF6 axis by GLA contributed to its antitumor effect on GC cells. These findings indicate that miR-3658 plays a tumor-suppressor role and participates in mediating the downregulation of MDM2/RNF6 by GLA. Given the critical role of miR-3658 in the GLA-MDM2/RNF6 mechanism, we then sought to examine how GLA affected the expression of miR-3658. Studies have demonstrated that DNA methylation plays a crucial role in silencing tumor-suppressive miRNAs in tumors (41–43). To determine whether suppression of DNA hypermethylation was involved in the upregulation of miR-3658 by GLA in the GC cells, 5-Aza-CdR, a widely used demethylation inducer, was utilized to treat GC cells. The levels of miR-3658 were significantly increased in the presence of 5-Aza-CdR. Thus, we deduced that suppression of miR-3658 in GC cells was mediated by hypermethylation that was then validated by anti-EZH2 and anti- H3K27me3 CHIP assays. These results suggest that GLA can “switch on” the transcription of miR-3658 *via* demethylation. Further studies are needed to examine whether GLA could play a general role of demethylation inducer in cancer cells by epigenetically “turning on” the transcription of hypermethylated tumor suppressor genes.

In addition to the miR-3658 pathway, our pathway analysis data in GC cells treated with GLA showed that GLA could activate the SMG1-UPF pathway that functions to downregulate the expression of various essential factors facilitating tumorigenesis by inducing mRNA decay (26, 27). The SMG1-UPF pathway is a core part of NMD, a quality-control mechanism that rapidly degrades aberrant mRNAs (22, 23). Impairment of NMD-mediated mRNA degradation against oncogenesis is involved in various pathophysiological processes of cancer (44–46). Tumor suppressor genes and factors are prone to induce NMD that can downregulate the expression of many oncogenes and factors implicated in signaling pathways that lead to cancer (44, 47). We found that GLA enhanced the mRNA decay of MDM2 and RNF6 *via* SMG1 pathway, thus to further decrease their expression at the posttranscriptional level. Based on the findings in our study, the SMG1-UPF mRNA decay pathway and miR-3658 silencing are two independent posttranscriptional regulatory mechanisms underlying the inhibitory effect of GLA on gastric cancer cells, and they have an additive effect of enabling the desired anti-cancer effect of GLA. However, more studies are needed to further validate the relationship between miR-3658 demethylation and SMG1-UPF mRNA decay induced by GLA, such as functional analysis, p53 degradation or protein ubiquitination.

Our results reveal that the activation of both miR-3658-mediated RNA silencing and the SMG1 mRNA decay mechanism that hampers the expression of oncogenes, *Mdm*2 and *RNF6*, contributes to the inhibitory effect of GLA on GC cells. Being a natural compound from Rab- dosia japonica var, GLA could greatly benefit treating GC by targeting the expression of oncogenes represented by MDM2 and RNF6 in a posttranscriptional dependent manner.

## Data Availability Statement

The original contributions presented in the study are included in the article/**Supplementary Material**. Further inquiries can be directed to the corresponding author.

## Ethics Statement

Animal experiments were performed according to the guidelines provided by the experimental animal center of Hangzhou Huqingyu Hall Pharmaceutical Co., Ltd, Hangzhou, China, and approved by the Animal Care and Use Committee of Hangzhou Huqingyu Hall Pharmaceutical Co., Ltd.

## Author Contributions

YL, PC, DQ, and LC conceived the project. DQ, YL, PC, and LC performed the study. PC, YL, DQ, and LC wrote the manuscript. PC supervised the project. All authors contributed to the article and approved the submitted version.

## Conflict of Interest

The authors declare that this study received funding from Hangzhou Huqingyu Hall Pharmaceutical Co., Ltd. The funder had the following involvement in the study: DQ and LC were involved in the study design, collection, analysis, interpretation of data, and the writing of this article.

The remaining authors declare that the research was conducted in the absence of any commercial or financial relationships that could be construed as a potential conflict of interest.

## Publisher’s Note

All claims expressed in this article are solely those of the authors and do not necessarily represent those of their affiliated organizations, or those of the publisher, the editors and the reviewers. Any product that may be evaluated in this article, or claim that may be made by its manufacturer, is not guaranteed or endorsed by the publisher.

## Supplementary Material

Supplementary Figure 1 Cell viability of GES-1 and Hs 738.St/Int cells treated with 0, 0.1, 0.5, 2.5, 5, and 10 µM of GLA at 24 h, 48 h, or 72h (% of control). The data represented the mean ± SD (n=3).

## References

[1] Machlowska J Baj J Sitarz M Maciejewski R Sitarz R. Gastric Cancer: Epidemiology, Risk Factors, Classification, Genomic Characteristics and Treatment Strategies. Int J Mol Sci (2020) 21(11):4012. doi: 10.3390/ijms21114012 PMC7312039 32512697

[2] Chen J Zhang W Pan C Fan J Zhong X Tang S. Glaucocalyxin A Induces Cell Cycle Arrest and Apoptosis *via* Inhibiting NF κb/P65 Signaling Pathway in Melanoma Cells. Life Sci (2021) 271:119185. doi: 10.1016/j.lfs.2021.119185 33577846

[3] Zhang C Ma K Yang YM Wang FQ Li WY. Glaucocalyxin A Suppresses Inflammatory Responses and Induces Apoptosis in TNF-A-Induced Human Rheumatoid Arthritis *via* Modulation of the STAT3 Pathway. Chem Biol Interact (2021) 341:109451. doi: 10.1016/j.cbi.2021.109451 33798506

[4] Zhu MS Shan J Xu HE Xia GM Xu Q Quan K. Glaucocalyxin A Suppresses Osteoclastogenesis Induced by RANKL and Osteoporosis Induced by Ovariectomy by Inhibiting the NF- κb and Akt Pathways. J Ethnopharmacol (2021) 276:114176. doi: 10.1016/j.jep.2021.114176 33933570

[5] Mao M Zhang T Wang ZY Wang HS Xu J Yin F. Glaucocalyxin A-Induced Oxidative Stress Inhibits the Activation of STAT3 Signaling Pathway and Suppresses Osteosarcoma Progression *In Vitro* and In Vivo. Biochim Biophys Acta Mol Basis Dis (2019) 1865:1214–25. doi: 10.1016/j.bbadis.2019.01.016 30659925

[6] Li M Chen C Wang Q Jiang X Tan L Huang Y. Glaucocalyxin A Suppresses Multiple Myeloma Progression *In Vitro* and *In Vivo* Through Inhibiting the Activation of STAT3 Signaling Pathway. Cancer Cell Int (2021) 21(1):683. doi: 10.1186/s12935-021-02375-z 34923957 PMC8684694

[7] Piao YH Jiang JZ Wang ZG Wang CY Jin S Li L. Glaucocalyxin A Attenuates Allergic Responses by Inhibiting Mast Cell Degranulation Through P38mapk/NrF2/HO-1 and HMGB1/TLR4/NF- κb Signaling Pathways. Evid Based Complement Alternat Med (2021) 2021:6644751. doi: 10.1155/2021/6644751 34007295 PMC8110394

[8] Peng Z Zhang R Pan LF Pei HH Niu ZQ Wang H. Glaucocalyxin A Protects H9c2 Cells Against Hypoxia/Reoxygenation-Induced Injury Through the Activation of Akt/Nrf2/HO-1 Pathway. Cell Transplant (2020) 29:963689720967672. doi: 10.1177/0963689720967672 33172292 PMC7784558

[9] Shi F Xue D Jiang Q Qiu J. Glaucocalyxin A Induces Apoptosis and Autophagy in Tongue Squamous Cells Carcinoma Cells by Regulating ROS. Cancer Chemother Pharmacol (2021) 88(2):235–46. doi: 10.1007/s00280-021-04285-3 33904969

[10] Sciot R. MDM2 Amplified Sarcomas: A Literature Review. Diagnostics (Basel) (2021) 11(3):496. doi: 10.3390/diagnostics11030496 33799733 PMC8001728

[11] Wang HZ Lu YD Wang ML Wu YL Wang XD Li YX. Roles of E3 Ubiquitin Ligases in Gastric Cancer Carcinogenesis and Their Effects on Cisplatin Resistance. J Mol Med (Berl) (2021) 99(2):193–212. doi: 10.1007/s00109-020-02015-5 33392633

[12] Konopleva M Martinelli G Daver N Papayannidis C Wei A Higgins B. MDM2 Inhibition: An Important Step Forward in Cancer Therapy. Leukemia (2020) 34:2858–74. doi: 10.1038/s41375-020-0949-z 32651541

[13] Timóteo M Tavares A Cruz S Campos C Medeiros R Sousa H. Association of Murine Double Minute 2 Polymorphisms With Gastric Cancer: A Systematic Review With Meta-Analysis. BioMed Rep (2021) 15(2):69. doi: 10.3892/br.2021.1445 34257965 PMC8243240

[14] Tang M Zeng X Luo J Quan FF Chen CY Li YK. Gene Commander in the Trash Heap: Transcriptional Regulation and Ubiquitination Modification Mediated by RNF6 in Carcinogenesis. Exp Cell Res (2021) 401(1):112396. doi: 10.1016/j.yexcr.2020.112396 33485842

[15] Huang Z Cai Y Yang C Chen Z Sun H Xu Y. Knockdown of RNF6 Inhibits Gastric Cancer Cell Growth by Suppressing STAT3 Signaling. Onco Targets Ther (2018) 11:6579–87. doi: 10.2147/OTT.S174846 PMC6178940 30323630

[16] Prinz C Mese K Weber D. MicroRNA Changes in Gastric Carcinogenesis: Differential Dysregulation During Helicobacter Pyloriand EBV Infection. Genes (Basel) (2021) 12(4):597. doi: 10.3390/genes12040597 33921696 PMC8073778

[17] Hosseini F Soltani BM Baharvand H Hosseinkhani S. Hsa-miR-3658 Down-Regulates OCT4 Gene Expression Followed by Suppressing SW480 Cell Proliferation and Migration. Biochem J (2020) 477(12):2281–93. doi: 10.1042/BCJ20190619 32478824

[18] Weidle UH Birzele F Nopora A. MicroRNAs Promoting Growth of Gastric Cancer Xenografts and Correlation to Clinical Prognosis. Cancer Genomics Proteomics (2021) 18(1):1–15. doi: 10.21873/cgp.20237 33419892 PMC7796821

[19] Wang J Sun Y Zhang X Cai H Zhang C Qu H. Oxidative Stress Activates NORAD Expression by H3K27ac and Promotes Oxaliplatin Resistance in Gastric Cancer by Enhancing Autophagy Flux via Targeting the miR-433-3p. Cell Death Dis (2021) 12(1):90. doi: 10.1038/s41419-020-03368-y 33462197 PMC7814071

[20] Supek F Lehner B Lindeboom RGF. To NMD or Not to NMD: Nonsense-Mediated mRNA Decay in Cancer and Other Genetic Disease. Trends Genet (2021) 37(7):657–68. doi: 10.1016/j.tig.2020.11.002 33277042

[21] Zhou X Ma W Li X Xu J. Glaucocalyxin A Prevents Hypoxia-Induced Epithelial-Mesenchymal Transition in Human Gastric Cancer Cells Through the PI3K/Akt Signaling Pathway. J Recept Signal Transduct Res (2020) 13:1–8. doi: 10.1080/10799893.2020.1853160 33307912

[22] Boresowicz J Kober P Rusetska N Maksymowicz M Paziewska A Dabrowska M. DNA Methylation Influences miRNA Expression in Gonadotroph Pituitary Tumors. Life (Basel) (2020) 10(5):59. doi: 10.3390/life10050059 PMC7281098 32413978

[23] Suzuki H Maruyama R Yamamoto E Kai M. DNA Methylation and microRNA Dysregulation in Cancer. Mol Oncol (2012) 6(6):567–78. doi: 10.1016/j.molonc.2012.07.007 PMC5528344 22902148

[24] Varghese VK Shukla V Kabekkodu SP Pandey D Satyamoorthy K. DNA Methylation Regulated microRNAs in Human Cervical Cancer. Mol Carcinog (2018) 57(3):370–82. doi: 10.1002/mc.22761 29077234

[25] Singh DK Bose S Kumar S. Regulation of Expression of microRNAs by DNA Methylation in Lung Cancer. Biomarkers (2016) 21(7):589–99. doi: 10.3109/1354750X.2016.1171906 27122255

[26] Nogueira G Fernandes R Garciía-Moreno JF Romão L. Nonsense-Mediated RNA Decay and Its Bipolar Function in Cancer. Mol Cancer (2021) 20:72. doi: 10.1186/s12943-021-01364-0 33926465 PMC8082775

[27] Sato H Singer RH. Cellular Variability of Nonsense-Mediated mRNA Decay. Nat Commun (2021) 12(1):7203. doi: 10.1038/s41467-021-27423-0 34893608 PMC8664836

[28] Manfredi JJ. Mdm2 and MdmX: Partners in P53 Destruction. Cancer Res (2021) 81(7):1633–4. doi: 10.1158/0008-5472.CAN-21-0145 34003788

[29] Aboushousha T Helal N Hammam O Ibrahim M Khaled S Mostafa A. Overview of MDM2 and B-RAF Expression in Gastric Lesions. Open Access Maced J Med Sci (2018) 6(10):1795–802. doi: 10.3889/oamjms.2018.338 PMC6236038 30455751

[30] Hou H Sun D Zhang X. The Role of MDM2 Amplification and Overexpression in Therapeutic Resistance of Malignant Tumors. Cancer Cell Int (2019) 19:216. doi: 10.1186/s12935-019-0937-4 31440117 PMC6704499

[31] Chibaya L Karim B Zhang H Jones SN. MDM2 Phosphorylation by Akt Regulates the P53 Response to Oxidative Stress to Promote Cell Proliferation and Tumorigenesis. Proc Natl Acad Sci USA (2021) 118(4):e2003193118. doi: 10.1073/pnas.2003193118 33468664 PMC7848548

[32] Li Q Wang G Tao J Chen W. RNF6 Promotes Colorectal Cancer Invasion and Migration *via* the Wnt/beta-Catenin Pathway by Inhibiting GSK3beta Activity. Pathol Res Pract (2021) 225:153545. doi: 10.1016/j.prp.2021.153545 34352441

[33] Huang Y Zou Y Jie Z. RNF6 Promotes the Migration and Invasion of Breast Cancer by Promoting the Ubiquitination and Degradation of MST1. Exp Ther Med (2022) 23(2):118. doi: 10.3892/etm.2022.11255 34970341 PMC8713179

[34] Cai J Xiong Q Jiang X Zhou S Liu T. RNF6 Facilitates Metastasis and Radioresistance in Hepatocellular Carcinoma Through Ubiquitination of Foxa1. Exp Cell Res (2019) 374(1):152–61. doi: 10.1016/j.yexcr.2018.11.019 30496760

[35] Ren Y Xu X Mao CY Han KK Xu YJ Cao BY. RNF6 Promotes Myeloma Cell Proliferation and Survival by Inducing Glucocorticoid Receptor Polyubiquitination. Acta Pharmacol Sin (2020) 41(3):394–403. doi: 10.1038/s41401-019-0309-6 31645658 PMC7470801

[36] Tursun B Schluter A Peters MA Viehweger B Ostendorff HP Soosairajah J. The Ubiquitin Ligase Rnf6 Regulates Local LIM Kinase 1 Levels in Axonal Growth Cones. Genes Dev (2005) 19:2307–19. doi: 10.1101/gad.1340605 PMC1240040 16204183

[37] Sun TS Liu ZN Yang Q. The Role of Ubiquitination and Deubiquitination in Cancer Metabolism. BMC Mol Cancer (2020) 19:146. doi: 10.1186/s12943-020-01262-x PMC7529510 33004065

[38] Liu X Ma R Yi B Riker AI Xi Y. MicroRNAs Are Involved in the Development and Progression of Gastric Cancer. Acta Pharmacol Sin (2021) 42(7):1018–26. doi: 10.1038/s41401-020-00540-0 PMC8208993 33037405

[39] Hussen BM Hidayat HJ Salihi A Sabir DK Taheri M Ghafouri-Fard S. MicroRNA: A Signature for Cancer Progression. BioMed Pharmacother (2021) 138:111528. doi: 10.1016/j.biopha.2021.111528 33770669

[40] Inoue J Inazawa J. Cancer-Associated miRNAs and Their Therapeutic Potential. J Hum Genet (2021) 66(9):937–45. doi: 10.1038/s10038-021-00938-6 34088973

[41] Pajares MJ Alemany-Cosme E Goni S Bandres E Palanca-Ballester C Sandoval J. Epigenetic Regulation of microRNAs in Cancer: Shortening the Distance From Bench to Bedside. In J Mol Sci (2021) 22(14):7350. doi: 10.3390/ijms22147350 PMC8306710 34298969

[42] Ma J Hong L Chen Z Nie Y Fan D. Epigenetic Regulation of microRNAs in Gastric Cancer. Dig Dis Sci (2014) 59(4):716–23. doi: 10.1007/s10620-013-2939-8 24248419

[43] Shin VY Chu KM. MiRNA as Potential Biomarkers and Therapeutic Targets for Gastric Cancer. World J Gastroenterol (2014) 20(30):10432–9. doi: 10.3748/wjg.v20.i30.10432 PMC4130850 25132759

[44] Lindeboom RGH Supek F Lehner B. The Rules and Impact of Nonsense- Mediated mRNA Decay in Human Cancers. Nat Genet (2016) 48:1112–8. doi: 10.1038/ng.3664 PMC5045715 27618451

[45] Liu C Karam R Zhou Y Su F Ji Y Li G. The UPF1 RNA Surveillance Gene Is Commonly Mutated in Pancreatic Adenosquamous Carcinoma. Nat Med (2014) 20:596–8. doi: 10.1038/nm.3548 PMC4048332 24859531

[46] Lu J Plank T-D Su F Shi X Liu C Ji Y. The Nonsense-Mediated RNA Decay Pathway Is Disrupted in Inflammatory Myofibroblastic Tumors. J Clin Invest (2016) 126:3058–62. doi: 10.1172/JCI86508 PMC4966300 27348585

[47] Mort M Ivanov D Cooper DN Chuzhanova NA. A Meta-Analysis of Nonsense Mutations Causing Human Genetic Disease. Hum Mutat (2008) 29:1037–47. doi: 10.1002/humu.20763 18454449

