# Supplementary figures and images for: Glaucocalyxin A Inhibits the Malignancies of Gastric Cancer Cells by Downregulating MDM2 and RNF6 via MiR-3658 and the SMG1-UPF mRNA Decay Pathway
Source: Front Oncol. 2022 Jun 22;12:871169. doi: 10.3389/fonc.2022.871169 (PMC9258495; doi:10.3389/fonc.2022.871169)

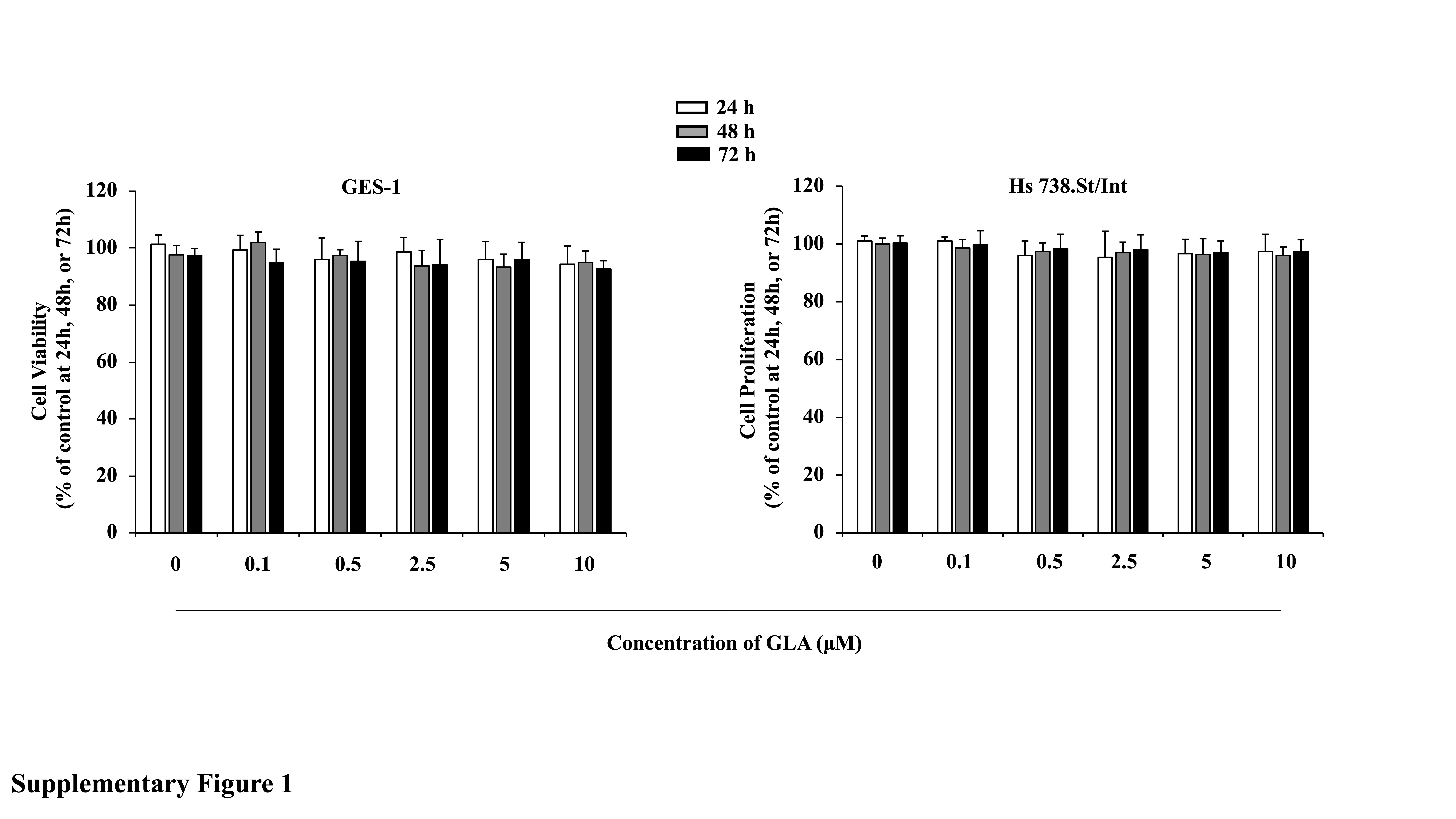

Supplement: Supplementary Figure 1 — Cell viability of GES-1 and Hs 738.St/Int cells treated with 0, 0.1, 0.5, 2.5, 5, and 10 µM of GLA at 24 h, 48 h, or 72h (% of control). The data represented the mean ± SD (n=3). [file Image_1.jpeg]
